# Spectacles and Eyeglasses; Their Forms, Mounting and Proper Adjustment

**Published:** 1903-10

**Authors:** 


Spectacles and Eyeglasses; Their Forms, Mounting and Proper Adjustment.—By R. J. Phillips, M. D., Opthalmologist Presbyterian Orphanage, late Adjunct Professor of Diseases of the Eye, Philadelphia Polyclinic and College for Graduates in Medicine, etc. Third Edition, revised, with 52 illustrations. Philadelphia: P. Blakiston's Son & Co., 1012 Walnut Street. 1902. Price, $1.00 net.

The purpose and scope of this excellent little manual is well stated in the author's preface, as follows: "The book, like the teaching referred to, is intended to supplement studies in refraction, and to give the student that knowledge of the correct placing of the glasses before the eye, without which the most painstaking measurements of the refraction will frequently fail of practical result." R. & L.

